# Farm management practices associated with influenza A virus contamination of people working in Midwestern United States swine farms

**DOI:** 10.1186/s40813-023-00304-2

**Published:** 2023-05-15

**Authors:** Gustavo Lopez-Moreno, Marie R. Culhane, Peter Davies, Cesar Corzo, Matthew W. Allerson, Montserrat Torremorell

**Affiliations:** 1 Department of Veterinary Population Medicine, College of Veterinary Medicine, University of Minnesota, Saint Paul, MN USA; 2 Holden Farms, Inc., Northfield, MN USA

**Keywords:** Influenza A virus, Pigs, Breeding herds, Internal biosecurity, Management practices

## Abstract

Indirect transmission of influenza A virus (IAV) contributes to virus spread in pigs. To identify farm management activities with the ability to contaminate farmworkers’ hands and clothing that then could be a source of virus spread to other pigs, we conducted a within-farm, prospective IAV surveillance study. Hands and clothes from farmworkers performing the activities of piglet processing, vaccination, or weaning were sampled before and after the activities were performed. Samples were tested by IAV rRT-PCR and virus viability was assessed by cell culture. A multivariate generalized linear model was used to detect associations of the activities with IAV contamination. Of the samples collected for IAV rRT-PCR testing, there were 16% (12/76) collected immediately after processing, 96% (45/48) collected after vaccination, and 94% (29/31) collected after weaning that tested positive. Samples collected immediately after vaccination and weaning, i.e., activities that took place during the peri-weaning period when pigs were about 3 weeks of age, had almost 6 times higher risk of IAV detection and had more samples IAV positive (*p*-value < 0.0001) than samples collected after processing, i.e., an activity that took place in the first few days of life. Both, hands and clothes had similar contamination rates (46% and 55% respectively, *p*-value = 0.42) and viable virus was isolated from both. Our results indicate that activities that involve the handling of infected piglets close to weaning age represent a significant risk for IAV dissemination due to the high level of IAV contamination found in farmworkers’ hands and coveralls involved in the activities. Biosecurity protocols that include hand sanitation and changing clothing after performing activities with a high-risk of influenza contamination should be recommended to farmworkers to control and limit the mechanical spread of IAV between pigs.

## Introduction

Influenza A virus (IAV) is endemic in pigs and one of the pathogens part of the porcine respiratory disease complex causing important economic losses to swine producers [1–3]. IAV can also cause zoonotic infections of pandemic potential [4–6] and one of the main concerns is the risk of bi-directional transmission of IAV at the pig-human interface. Such transmission events have resulted in infections in farmworkers [7], the general public attending agricultural fairs [8] and patrons at live animal markets [9]. In addition, events where people have infected pigs are also relevant [10] as the introduction of human-origin IAV into pigs is considered one of the main contributors of IAV genetic diversity found in pigs [11] making control of influenza difficult. As a result, pigs can become reservoirs for IAV strains that can infect people. Overall, there is a need to control IAV in pigs and decrease the risk of bi-directional transmission between pigs and people.

A starting point to control IAV in pigs is preventing infections of piglets before weaning. Piglets are born naïve to IAV, they can become infected very early in life [1] and swine herds can sustain IAV infections for prolonged periods of time. The on-going birth of piglets and the recruitment of these naïve, neonatal pigs into the infection chain occurring in breeding herds facilitates IAV endemicity [12]. At weaning, pigs can be transported to distant locations resulting in the spread of IAV strains into new geographical regions [13]. Ensuring that piglets are weaned IAV negative is appealing to swine producers because of the benefits afforded towards pig health, productivity and well-being since IAV negative pigs have less respiratory disease and grow faster and more efficiently.

From the moment piglets are born, piglets have close interactions with farmworkers. Piglets may be handled during the birthing process and shortly after that to dry them, ensure colostrum intake, prevent injuries, apply iron injections, clip teeth, conduct castration and vaccination, and possibly piglets may be moved into other litters. Some of the management practices, such as cross-fostering and use of nurse sows, in place during this period have been shown to facilitate the spread of IAV among the piglets [14, 15]. Furthermore, the transmission of IAV via fomites including contaminated personal protective equipment (PPE) worn by personnel working with pigs has been shown experimentally [16], and transmission could take place in the presence of both, basic and enhanced biosecurity practices. Although the use of PPE (i.e., gloves and face masks) is usually recommended, its use by farmworkers is not widely implemented [17]. Even if workers use gloves while handling pigs, the gloves may not be changed when they switch between chores or when workers are moving between different areas of the farm.

Implementation of biosecurity practices directed at preventing the spread of IAV among litters, such as changing gloves between litters when handling pigs, no use of nurse sows or cross-fostering, can delay IAV infections in piglets [18]. However, these practices, as stand-alone protocols, do not seem to be sufficient to fully prevent the spread of IAV among litters. Detection of viable IAV in hands of farmworkers implementing these practices and in materials used to handle piglets has also been documented [16, 18]. Thus, we hypothesize that there are specific, high-risk management practices involving the handling of piglets that facilitate the contamination of farmworkers’ hands and clothes that in turn, may become a source of IAV infection to other piglets. Thus, understanding high-risk management practices occurring while handling pigs during the pre-weaning period can help in the control of IAV in pigs. The objective of our study was to investigate the association of specific management practices that require intensive handling of pigs (i.e., processing, vaccination and weaning) with risk of IAV contamination of farmworkers’ hands and clothes. Results from this study can add to biosecurity recommendations to swine producers to limit the mechanical transmission of IAV in pigs.

## Materials and methods

### Experimental design

Four breeding herds that belonged to the same production company and were part of an IAV surveillance program were conveniently selected for the study. The breeding herds had a history of IAV infection in weaned pigs and their IAV status was confirmed as part of the enrollment criteria. Herd selection criteria consisted of the producer’s willingness to participate in the study and the researchers’ ability to sample litters and hands and clothes of farmworkers when performing farm chores. IAV herd status was confirmed by collecting thirty udder wipe samples [19] from litters of weaning age to detect at least one positive litter when the IAV prevalence was at least 10% with a 95% confidence interval. Farms where IAV was not detected as part of this initial sampling were excluded from the study. Before initiating the study, researchers selected activities in the pre-weaning period to determine if they were likely to result in a high-risk of IAV contamination. These activities required handling of pigs and included processing, vaccination, and weaning.

Processing of piglets occurs at about 3–5 days of age, is done on multiple litters a day and usually it requires farmworkers to place all the piglets from the litter together into a cart to facilitate handling. During processing, piglets are administered iron intramuscularly, male piglets are castrated, tails are docked to prevent tail biting and, in some farms, teeth are clipped to prevent injuries from fighting. Once processing is done, piglets are returned to their crate and the farmworkers move on to process the next litter. Most farms have protocols for disinfecting or discarding the teeth-clipper (e.g., nippers), tail-docker (e.g., clippers), castration materials (e.g., bisturi blades) and needles after a certain number of litters, but other materials such as carts and syringes are only cleaned and disinfected at the end of the day. Similarly, most workers use latex gloves for processing; however, these may not be changed between litters or only changed between few litters as indicated in the  farm internal biosecurity protocols [20].

Vaccination is an important disease preventive measure to protect piglets from infections caused by agents such as porcine circovirus type 2, porcine reproductive and respiratory syndrome virus (PRRSV) and *Mycoplasma hyopneumoniae*. Vaccination may occur at various ages during the pre-weaning period but commonly occurs around 16–20 days of age. Similar to processing, multiple litters are vaccinated on the same day. This requires piglets to be handled manually, usually by a single farmworker who lifts the piglet and secures it in his/her arms while injecting the vaccine into the neck muscles behind the pig’s ear. Depending on the piglet’s age, a second farmworker may assist in the vaccination process. Once the piglet is vaccinated, the pig is returned to its crate and after all the piglets in the crate have been vaccinated, the farmworkers move on to the next litter to repeat the process. In general, there are no protocols for changing gloves or clothing between litters after vaccination. Disposable gloves may be discarded at the end of the activity but farmworkers will usually wear the same clothes after performing the activity.

Lastly, weaning refers to the process of separating the piglets from their dam once piglets are ready to consume solid food. The timing of weaning varies between farms, but in North America, it usually takes place between 19 and 24 days of piglet’s age [21]. Multiple litters are weaned simultaneously within entire rooms and several rooms may be weaned on a single day on large farms of  > 1000 number of sows. This activity requires multiple farmworkers to lift the separation boards from the crates and, using sorting boards, moving the piglets in direction of the load-out chute then into a truck that will take them to a nursery or wean-to-finish site. Weaning usually occurs first thing in the morning and in most cases there are no protocols for changing gloves or clothing after the activity is finished. Occasionally, farmworkers will discard the disposable gloves if worn.

### Sample collection

To monitor the IAV status of the participating farms during the study, ninety udder skin wipes were collected from lactating sows at weaning. Udder skin wipes collect piglet’s nasal and oral secretions deposited during suckling on the sow’s udder skin. Samples are collected with a moistened wipe that includes cell culture media. The use of udder skin wipes has been described as a cost-efficient and sensitive method to detect IAV in breeding herds [19].

Workers were requested to wash their hands thoroughly with water and soap before initiating the activities of processing, vaccination or weaning. Workers were then provided with new PPE consisting of disposable coveralls (DuPont^tm^ Tyvek®, Wilmington, Delaware, USA) and a pair of latex gloves to be worn while performing the activities. A member of the research team was present at the farm to provide the PPE, to observe the workers while conducting the activities, and to sample the hands and coveralls of the workers immediately before and after the activities. The activities were performed following the already established farm protocols as described above.

**Fig. 1:**
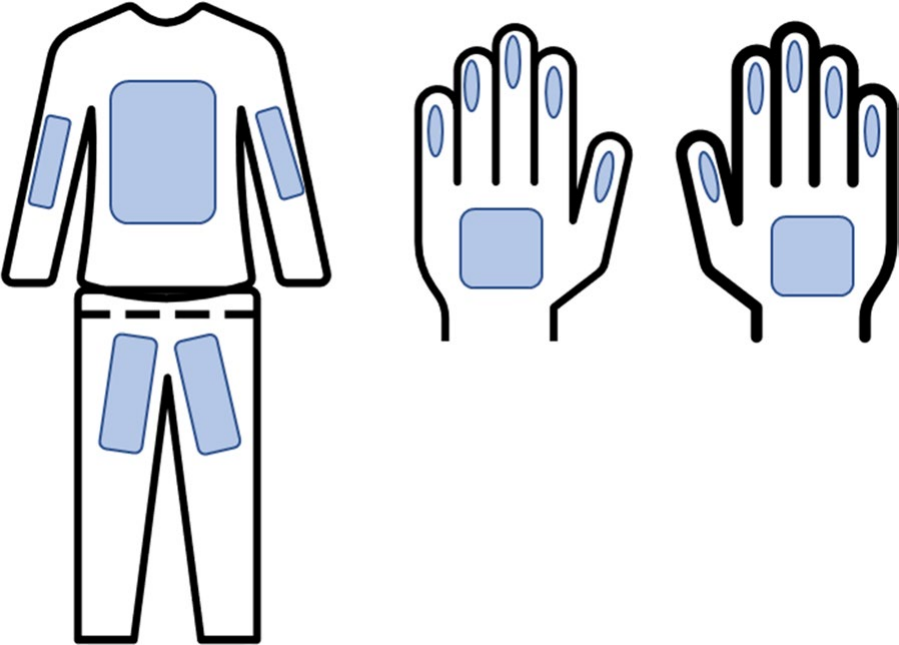
Illustration of the areas from coveralls and hands from which samples were collected

Samples from hands and coveralls were collected by wiping thoroughly pre-designated surfaces of the hands and coveralls using a wet gauze. An area of approximately 30 cm height × 30 cm width from the coverall with direct contact with the piglets which included arms, chest, and groin area was sampled. Both palmar areas from the hands and fingers were also sampled (Fig. 1). After collection, the samples were placed in an individually identified bag with transport media DMEM-Dulbecco’s Modified Eagle Medium Gibco™(Grand Island, NY, USA) supplemented with 0.5 ml of Gentamicin Sulfate (BioWhittaker®, Walkersville, MD USA) and 5 ml of antibiotics and antimycotic Anti-Anti (100×) Gibco™(Grand Island, NY, USA). Samples were kept refrigerated at 4 °C during transportation to the University of Minnesota laboratories, where aliquots of 2 ml were made and frozen at − 80 °C until testing was performed.

### Sample testing

All samples were processed for viral RNA extraction using a magnetic particle processor procedure (Ambion® MagMAX™AM1835, Viral RNA Isolation Kit; Applied Biosystems, Foster City, CA, USA). Samples were tested by rRT-PCR targeting the highly conserved IAV matrix gene, following previously described procedures [22]. Samples with cycle threshold (Ct) values lower than 35 were considered positive and those which Ct values equal or higher than 35 were considered negative. In order to confirm virus viability, a subset of 47 rRT-PCR positive samples were further tested for virus isolation using Madin-Darby canine kidney (MDCK) cells [23]. Sample selection ensured that all activities and sample types were included with preference to those with lowest Ct values to maximize the likelihood of viral isolation. MDCK cells were prepared in six-well plates that were inoculated with the selected samples and incubated for one hour at 37 °C with 5% CO_2_. Viral growth media was then added to each well that consisted of DMEM-Dulbecco's Modified Eagle Medium supplemented with 7.5% bovine serum albumin, antibiotics and antimycotic (Gibco™Grand Island, NY, USA) and trypsin-TPCK. The wells were inoculated in duplicates, with 200 μl and 100 μl of each sample and incubated for 1 h at 37 °C at 5% CO_2_. Plates were evaluated at day 3 and 5 for appearance of positive cytopathic effects (CPE). Samples were considered positive when cytopathic effect at day 5 post inoculation was observed and was confirmed positive using an hemagglutination assay with 20% turkey red blood cells [24].

### Statistical analysis

Data collected from the rRT-PCR results were consolidated in a spreadsheet (Microsoft EXCEL, Microsoft Corporation, Redmond, Washington, USA) and organized for analysis. Frequency counts and percentages were calculated for descriptive analysis. An exploratory analysis was completed to determine the differences in proportions of rRT-PCR sample results between sample types (hands and coveralls) and farm activities (processing, vaccination and weaning) using Pearson’s Chi-square test. Differences in the cycle threshold (Ct) obtained by rRT-PCR between sample type and farm activities were evaluated using a linear model followed by a pairwise t-test with a Bonferroni adjustment for multiple comparisons. To identify activities with increased risk of IAV detection and calculate prevalence ratio, a multivariate generalized linear model using R statistical software (version 4.1.1) was used [25]. IAV detection by rRT-PCR was included as the outcome variable. Sample type, farm activity and farm identification were added as predictor variables.

## Results

**Fig. 2:**
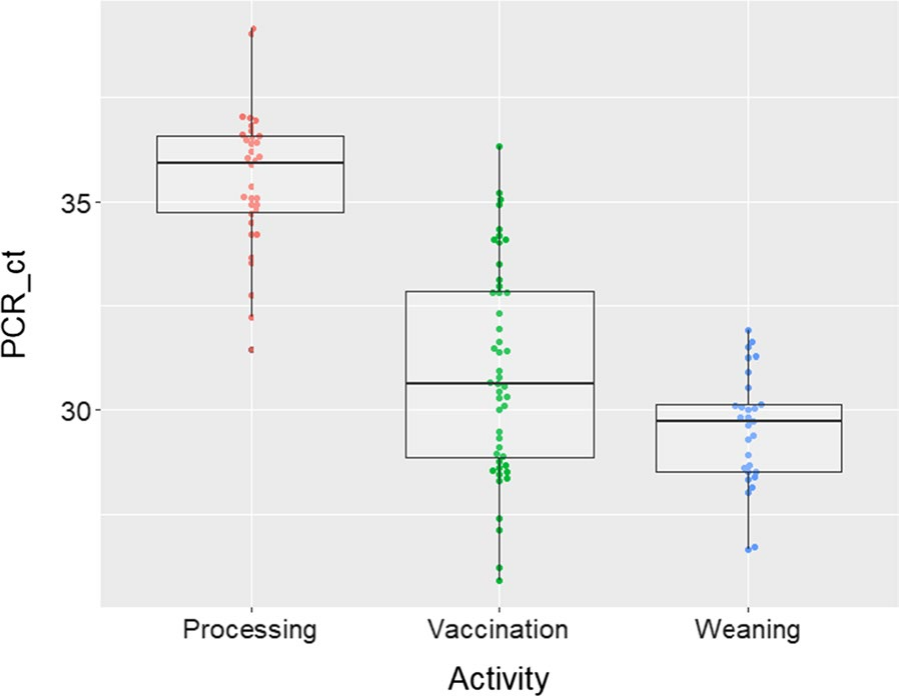
Distribution of influenza A virus cycle threshold (Ct) values obtained by rRT-PCR from samples collected from farmworkers’ hands and coveralls by activity. A Ct value lower than 35 was considered positive

From the four farms that were screened for IAV infections in suckling piglets, three were IAV positive and were kept in the study. Samples were collected in the months of December 2019, January and February 2020. The samples collected from litters during the course of the study showed that farm A had an IAV prevalence of 68.8% (62/90), farm B 34.4% (31/90) and farm C 81.1% (73/90). To ensure that farmworkers’ hands and the provided coveralls were not contaminated with IAV, we sampled them prior to initiating the activities with all 17 samples collected resulting in IAV rRT-PCR negative results. There were 155 samples collected immediately after the activities were concluded of which 75 were collected from farmworkers’ coveralls and 80 from their hands. Seventy-six samples were collected immediately after processing with 12 (15.8%) of them testing IAV rRT-PCR positive, nine from coveralls and 3 from hands (Table 1). Forty-eight samples were collected after vaccination of piglets and 45 (93.8%) of them were positive to IAV. All coveralls (19/19; 100%) were IAV positive after vaccination as were the majority (26/29; 89.7%) of the hand samples. From the weaning activity, 31 samples were collected and 29 (93.5%) of them tested IAV positive, 14 (14/15, 93.3%) from farmworkers’ hands and 15 (15/16, 93.8%) from their coveralls. Viable IAV was isolated from five samples and four of these samples had been collected after piglet vaccination (three from hands and one from coveralls) and one from a coverall at weaning. The Ct values obtained by rRT-PCR for samples collected after processing ranged from 31 to 39. The Ct values from samples collected after piglet vaccination ranged from 26 to 36 and the ones from weaning, ranged between 26 and 31 (Fig. 2). Differences in the Ct values obtained from the different activities were significant (*p*-value < 0.01) with weaning having the lowest mean Ct values.

**Table 1:** Number (percentage) of influenza A virus samples that tested rRT-PCR positive by farm and activity performed

Farm	Processing			Vaccination			Weaning		
	Hands (%)	Coverall (%)	Total (%)	Hands (%)	Coverall (%)	Total (%)	Hands (%)	Coverall (%)	Total (%)
A	1/14* (7.1)	3/15 (20)	4/29 (13.8)	13/16 (81.3)	8/8 (100)	21/24 (87.5)	7/8 (87.5)	6/6 (100)	13/14 (92.9)
B	0/5 (0)	0/5 (0)	0/10 (0)	5/5 (100)	3/3 (100)	8/8 (100)	1/1 (100)	3/3 (100)	4/4 (100)
C	2/17 (11.8)	6/20 (30)	8/37 (21.6)	8/8 (100)	8/8 (100)	16/16 (100)	6/6 (100)	6/7 (85.8)	12/13 (92.3)
Total	3/36 (8.3)	9/40 (22.5)	12/76 (15.8)	26/29 (89.7)	19/19 (100)	45/48 (93.8)	14/15 (93.3)	15/16 (93.8)	29/31 (93.5)

*Number of positive wipes/total number of wipes tested (percentage)

Samples were considered positive when the cycle threshold (Ct) value obtained by RT-PCR was below 35

There were significant differences in IAV positivity at processing compared with vaccination and weaning. Samples collected immediately after vaccination and weaning had approximately 6 times higher likelihood of testing IAV positive than samples collected after processing, with prevalence ratios of 6.20 and 5.98, for vaccination and weaning, respectively (*p*-value < 0.0001). However, there was no significant differences in IAV detection between farmworkers’ hands and coveralls (*p*-value = 0.42) (Table 2).

**Table 2:** Adjusted influenza A virus (IAV) positive proportions and prevalence ratio

Variable	Adjusted IAV positive proportion	Standard error	Prevalence ratio (95% CI)	*P*-value
Processing	0.15	0.04	Reference	–
Vaccination	0.94	0.15	6.20 (5.91–6.49)	< 0.0001
Weaning	0.91	0.18	5.98 (5.62–6.33)	< 0.0001
Hands	0.46	0.08	Reference	–
Coverall	0.55	0.10	1.19 (0.99–1.39)	0.42
Farm A	0.49	0.09	Reference	–
Farm B	0.48	0.14	0.97	0.99
Farm C	0.56	0.10	1.13	0.86

Adjusted influenza A virus (IAV) positive proportions and prevalence ratios obtained from a generalized mixed regression model to evaluate the associations between farm activities and IAV detection in farmworkers’ hands and coveralls. Influenza A virus rRT-PCR detection was used as a dependent variable with sample type, activity performed, and farm identification as predictor variables

## Discussion

Contaminated fomites can play a role in the overall spread of IAV in animal [26] and people populations [27, 28]. To the knowledge of the authors, this is the first study that reports IAV contamination rates of farmworkers’ hands and clothing based on pig handling activities performed at the farms. Our results indicate that activities that involve handling of piglets before weaning and that require close contact between farmworkers and pigs likely represent a significant risk for IAV dissemination during the preweaning period.

Previous studies reported that piglets are a high-risk population for IAV infections in breeding herds [29]. Because of the piglet’s size and age, activities performed in the piglets often require manual restraint of the piglets and close contact between the farmworkers and piglets. We documented that vaccination and weaning, when the pigs are a few weeks of age, represent activities that result in a higher risk, almost sixfold, of IAV contamination of farmworkers’ hands and clothes compared to processing, which is an activity done on the first few days of life. The increased risk of activities conducted during the peri-weaning period is likely the result of pigs at that age having higher IAV infection rates than younger pigs [29]. IAV prevalence of up to 100% has been reported in pigs before weaning compared to less than 1% from pigs of about 1 week of age [30]. Furthermore, IAV can be found in high quantity in nasal and oral secretions of infected pigs [31], and given the pig’s nose anatomy and behavior, it should not come as a surprise that surfaces in contact with the pig’s nose and mouth can become contaminated relatively easily with IAV. As a result, infected pigs are a source of contamination to the workers handling them, and given the contagiousness of IAV, it is plausible that the contamination of farmworkers’ hands and clothing is enough to continue the infection chain to infect susceptible piglets. This is further supported by the fact that we were able to obtain viable virus from hands and clothes contaminated during vaccination and weaning-associated activities.

Vaccination in piglets requires that farmworkers hold each pig individually to apply the vaccine. Thus contamination of the farmworker performing the vaccination is very likely to occur and in turn makes the farmworker a potential source of IAV to susceptible piglets when they are being handled. Similarly, we found high contaminations rates from the samples collected at weaning. Although this is not an activity that requires the extensive piglet handling as vaccination does, it still requires close contact between farmworkers and piglets and their environment. Weaning involves handling of materials (i.e., crate separators, sorting boards) in direct contact with the piglets being weaned as they are loaded onto a truck. In contrast, detection of IAV in farmworkers’ hands and coveralls during processing was relatively low, although contamination rates were higher than anticipated and we also consider this finding of importance.

Piglets are born IAV naïve and processing is an activity that occurs early in the piglets’ life (~ 3–5 days). Therefore we were not expecting to obtain many or any IAV positive samples collected at this time point. However, our results indicated that there is already some degree of IAV contamination in piglets before processing. Our findings are in agreement with a previous study where a low prevalence (1.1%) of IAV infected piglets was reported in litters of 2 days of age [18]. Some piglets are likely getting infected before processing when they are handled during the birthing process in activities such as drying, litter balancing and bottle feeding, assuming that the piglets are handled with contaminated materials or if the farmworkers’ hands and clothes are contaminated. This is plausible since farmworkers performing such activities may not have protocols in place that require changing coveralls, using disposable gloves, and/or washing hands when assisting newborn piglets.

While fomite-mediated IAV spread from farmworkers’ clothing and hands to piglets is the most likely cause of IAV transmission, it is also possible that newborn litters are being infected because they are adopted by nurse sows with an IAV contaminated udder [14]. Nurse sows are lactating sows that have weaned their own litter and are used to adopt piglets at risk of falling behind or dying. However, nurse sows are more commonly used to adopt pigs of 5–10 days of age, thus it is not likely that the infection in the litters at processing originated from nurse sows. Other sources of contamination may be possible including the environment itself [32] although the pigs were born in rooms that had been cleaned and disinfected (C&D) between farrowing groups and an effective C&D would decrease the likelihood of the environment being the source of infection. Another potential source of contamination is workers infected with seasonal influenza [11]. However, although such infection is possible we do not think it played an important role in this study in part due to the widespread detection of IAV in the fomites. IAV infection from workers most likely will be limited to one or few employees at once and it is not likely to result in widespread contamination of surfaces like the ones sampled in the fomites of this study. It was outside the scope of the study to investigate the source or directionality of infection to newborn piglets. Overall our results indicate that contaminated hands and clothes are the most likely source of IAV spread to newborn piglets.

We also showed that contamination of farmworkers’ hands and clothes happens relatively easily when handling IAV infected piglets and that both hands and coveralls can potentially be a source of virus spread. Farm practices encourage hand washing during the day to limit the transmission of pathogens between litters and between pigs and farmworkers [33]. In addition, some farms have protocols that require changing gloves after handling a certain number of litters to decrease the risk of transmission of diseases such as PRRSV [34]. However, frequent hand washing or changing of disposable gloves is not always possible due to the extensive litter handling required daily and the lack of hand washing stations in farrowing areas [35]. In contrast, change of farm clothing during the working day is not common and most farms do not have recommendations for changing coveralls after conducting certain activities that favor disease spread. An outcome of our study could be to recommend changing coveralls after performing vaccination and weaning, or other activities that require close handling of pigs before initiating a new activities. Overall, having internal biosecurity protocols that include hand washing, changing disposable gloves, and changing clothing after performing activities with a high-risk of disease transmission is recommended. These are simple low-cost interventions that can have a significant impact on IAV transmission. Given the economic cost that IAV can inflict on swine production, these interventions can easily be justified. However, a comprehensive cost–benefit analysis may be needed when implementing more extensive biosecurity practices [18, 36].

In summary, we showed that activities that involve handling of piglets during the peri-weaning period result in high levels of IAV contamination of the farmworkers’ hands and coveralls which represents a risk for mechanical transmission of IAV between litters. We also showed evidence of IAV contamination of hands and clothes after performing activities that involve the processing of newborn piglets, which highlights the need to enhance internal biosecurity measures beginning at the moment piglets are born. Overall, our results can be used to provide science-based recommendations to improve management protocols directed at limiting the transmission of pathogens in pigs before weaning. The recommendations, if successfully implemented on farms, should assist in the control and elimination of IAV in pigs, and ultimately should help decrease the risk of IAV transmission to people.

## Data Availability

Data and materials are available upon request.
